# Clinical evaluation of Major Depressive Disorder (MDD) and Borderline Personality Disorder(BPD) in youth adolescents

**DOI:** 10.1192/j.eurpsy.2022.834

**Published:** 2022-09-01

**Authors:** V. Kaleda, E. Krylova, A. Kuleshov, A. Beburishvili

**Affiliations:** FSBSI «Mental Health Research Centre», Department Of Youth Psychiatry, Moscow, Russian Federation

**Keywords:** major depressive disorder, nonsuicidal self-injury, suicidal behaviour, borderline personality disorder

## Abstract

**Introduction:**

According to international studies, at least 70% of BPD in adolescence are comorbid with MDD.

**Objectives:**

To determine the clinical and psychopathological markers of MDD in BPD in adolescence.

**Methods:**

Clinical psychopathological interview, SCID-II, Hamilton Depression Rating Scale (HDRS). Sample: N=73 male and female, age: 18-25 with MDD and BPD.

**Results:**

MDD comorbid with BPD in adolescence is characterized by polymorphism of its’ psychopathological manifestations due to the structure of BPD and the input of the age factor. In the studied sample 31 (42,5%) with both MDD and BPD also revealed addictions; 24 patients (32,9%) - anxiety and obsessive-compulsive (OCD) disorders, 18 patients (24,7%) had overvalued ideas. The high contingency of MDD with autoaggressive actions confirmed their high suicidal risk (53 patients). Among them - 31 patients (58,5%) - had non-suicidal self-harm (NSSI), 7 patients – (13,2%) had suicidal attempt (SA), and 15 patients (28,3%) had NSSI and suicidal attempts (NSSI+SA). The highest incidence of NSSI and NSSI+SA was noted in MDD with addictive disorders: NSSI – N=20 (80,00%), NSSI+SA - N=5 (20,00%), SA – N=0. MDD with BPD and anxiety disorders: NSSI – N=9 (56,25%), NSSI+SA – N= 6 (37,50%), SA–N=1 (6,25%). MDD with BPD and overvalued ideas: NSSI – N=2 (16,67%), NSSI +SA – N= 4 (33,33%), SA N=6 (50,0%), (results p<0.01).

**Conclusions:**

Psychopathological relations between BPD and MDD in youth are different due to additional comorbid conditions, like addictions, anxiety and OCD, overvalued ideas and have clinical implications in terms of suicidal and NSSI risks, individualized interventions and prognosis.

**Disclosure:**

No significant relationships.

